# Outcomes Following Vascularised Bone Graft for the Treatment of Upper Limb Osteomyelitis: A Systematic Review and Meta-Analysis

**DOI:** 10.7759/cureus.94697

**Published:** 2025-10-16

**Authors:** Alex L Starkey, Mantaran S Bakshi, Harsimran Kaur Surinder Singh, Sanjana A Shetty, Osamah Al-Zubaidi, Leto A Warie, Ahmed El Shinnawi, Batool E Kareem, Samuel Teklay, Abdal Q Zafar

**Affiliations:** 1 Trauma and Orthopaedics, Royal Lancaster Infirmary, Lancaster, GBR; 2 Spinal Surgery, Southmead and Bristol Children's Hospital, Bristol, GBR; 3 General Surgery, St. Helier Hospital, London, GBR; 4 Ear, Nose, and Throat, Queen Alexandra Hospital, Portsmouth, GBR; 5 Plastic Surgery, Castle Hill Hospital, Hull, GBR; 6 Oral Maxillofacial Surgery, Hull Royal Infirmary, Hull, GBR; 7 Emergency Medicine, Queen's Hospital, Romford, GBR; 8 Emergency Medicine, Tunbridge Wells Hospital, Kent, GBR; 9 Plastic Surgery, Pinderfields Hospital, Wakefield, GBR; 10 Trauma and Orthopaedics, Whittington Hospital, London, GBR

**Keywords:** autologous bone graft, free vascularised fibular graft, osteomyelitis treatment, plastic and reconstructive surgery, upper limb reconstruction, upper limb surgery, vascularised bone grafting, vascularized bone graft

## Abstract

Osteomyelitis is a debilitating condition with treatment options ranging from antibiotics to surgical management. In cases where debridement of necrotic or infected tissue results in extensive bone and soft-tissue loss, creating a poorly vascularised cavity prone to persistent infection and mechanical instability, vascularised bone grafts (VBGs) can provide definitive reconstruction. This systematic review and meta-analysis aimed to assess outcomes of VBGs for upper limb osteomyelitis. A systematic search of electronic databases identified 330 papers related to VBGs, of which 10 retrospective case series met the inclusion criteria, cumulatively including 70 patients. Statistical analysis was performed using Open MetaAnalyst software (Center for Evidence-Based Medicine, Brown University, Providence, RI, US) with a random-effects model and 95% confidence intervals. The mean time to bony union post-VBG was 6.2 months, with 59 (87.7%) patients achieving union. Secondary surgery was required for 19 (26.1%) patients, and postoperative infection recurrence occurred in three (5.1%). Postoperative stress fracture was reported in one case with an overall incidence of 5.5%. These findings indicate that VBGs are an effective treatment for upper limb osteomyelitis, yielding high rates of bony union and a low incidence of complications. Compared with other treatment modalities, patients undergoing VBGs may benefit from shorter antibiotic courses and improved outcomes. Whilst the included studies were limited to retrospective series, this pooled analysis provides evidence supporting the use of VBGs as a reliable surgical option for complex upper limb osteomyelitis.

## Introduction and background

Osteomyelitis (OM) refers to a bacterial infection of bone, commonly caused by *Staphylococcus aureus* and related species [1,2]. It is a significant and debilitating complication of surgery and trauma, with often costly treatment regimens that place significant demand on healthcare services [3,4]. Compared with acute OM (which typically develops over days or weeks), established colonisation of bacteria in chronic OM can cause significant local tissue damage over months to years [5,6]. This condition can often occur secondary to the implantation of metalwork for fracture fixation and can compromise functional outcomes. Overall, approximately 5% of fracture fixation devices become infected, with rates as high as 50% following treatment of severe open fractures [7,8].

Upper limb OM is traditionally managed using a multidisciplinary approach. Under the aegis of a surgeon, microbiologist/infectious disease specialist, radiologist, and rehabilitation team, an individually tailored approach can lead to desirable outcomes in a difficult scenario. Mechanical and chemical debridement, along with targeted antimicrobial therapy, is the mainstay of management. This includes isolating the organism(s) and obtaining targeted antibiotic therapy, along with staged surgery to debulk and reduce the infection burden locally and prepare to provide a healthy vascular tissue bed for definitive bony union.

Medical management alone frequently fails to definitively treat OM, particularly in cases where implanted foreign bodies are the source of or reservoir for bacterial infection [9]. Several types of bacteria can establish three-dimensional matrices, known as biofilms, which are highly effective at resisting antibiotic therapy [7]. For this reason, surgical debridement and removal of metalwork are often the only appropriate treatments. In cases requiring extensive debridement of infected tissue, large bone defects are an adverse sequelae that are challenging to reconstruct.

Several modalities are available for bone defect reconstruction. A vascularised bone graft (VBG) may be used to bridge dead space, and in cases where tissues have been devitalised by concomitant OM, VBGs can improve the likelihood of successful healing [10-15]. The use of prevascularised tissue can safeguard against ischaemia and subsequent failure, which is particularly important in patients who may have already undergone multiple previous procedures. Common sites for VBG harvesting include the lateral femur, iliac crest, and fibula. The fibula is a particularly useful harvest site for upper limb defect grafting, given its long length and well-sized vascular pedicle [11,16]. Surgeries may be performed as single-stage or multiple-stage procedures, with debridement occurring either before or at the same time as graft implantation [17]. The fibula can also be harvested piecemeal, protecting each vascular pedicle to fill in multiple sites of defects, especially in areas of precarious blood supply and chronic scarring.

Across the literature, there are multiple reported cases of patients with chronically infected limb defects undergoing successful treatment with VBGs [16-25]. To date, there has been no comprehensive synthesis of outcomes examining the effects of VBGs for upper limb OM. The authors aimed to address the current void in the literature by performing a systematic review and meta-analysis to produce a thorough evaluation of the advantages and limitations of VBGs in the treatment of upper limb OM.

## Review

Methodology

This systematic review and meta-analysis was performed according to the Preferred Reporting Items for Systematic Reviews and Meta-Analyses (PRISMA) statement standards [26].

Inclusion Criteria

All studies, including observational, case-control, randomised, and nonrandomised trials, that reported outcomes following VBGs for upper limb OM were included. Only studies reporting three or more patient cases were included. There were no restrictions on patient age, sex, or comorbidities. Papers including both upper and lower limb cases were included, but only upper limb cases were extracted for the analysis. ‘Upper limb’ was defined as the three long bones of the upper limb (humerus, radius, and ulna), the wrist, or the hand. International studies were included if they provided an official English transcript of the original publication.

Exclusion Criteria

Studies reporting on two or fewer cases, or lacking an official English transcript in the original publication, were excluded.

Outcome Measures

The primary outcomes included time to union, incidence of union, infection recurrence, and occurrence of stress fractures after bone grafting. Secondary outcomes included the incidence of patients requiring further surgical procedures after VBG and the microbial profile of infections across the reported cases.

Literature Search Strategy

Authors ALS and MSB performed independent searches of MEDLINE, Google Scholar, PubMed, the Cumulative Index to Nursing and Allied Health Literature (CINAHL), the Central Register of Controlled Trials (CENTRAL), the World Health Organisation’s International Clinical Trials Registry, ClinicalTrials.gov, and the ISRCTN Database. The searches were concluded on February 23, 2024. The search terms used to screen the databases were ‘vascularised bone grafts’, ‘free fibula’, ‘medial femoral condyle flap for upper limb’, ‘forearm’, ‘osteomyelitis’, and ‘infection’. Adjuncts of ‘AND’ and ‘OR’ were used to collate the search terms. The bibliographies of relevant results were also searched to identify additional eligible studies.

Selection of Studies

Both ALS and MSB independently screened the abstracts and titles to compile a preliminary list of studies that met the inclusion criteria, and the full texts were then screened. Author AQZ was consulted to reach an agreement in the case of any selection discrepancies.

Data Extraction

An Excel spreadsheet (Microsoft Corp., Redmond, WA, US) was used to collect data based on the Cochrane data collection tool [27]. The information collected included the year of publication, number of patients, time to union, incidence of union, infection recurrence, incidence and subtype of further procedures, and the microbiological spectrum of the infection. All authors collaborated on data extraction, with ALS performing the final verification. Any highlighted discrepancies were rectified after a collective discussion.

Risk of Bias Assessment

The Newcastle-Ottawa scale (NOS) was used to assess the risk of bias within the included studies [28]. Star-rated domains include selection, compatibility, and outcomes, with a greater number of stars representing better methodological quality.

Statistical Analysis

Data synthesis was performed using Open MetaAnalyst software (Center for Evidence-Based Medicine, Brown University, Providence, RI, US). A random-effects model was applied for all meta-analyses to account for anticipated clinical and methodological heterogeneity amongst the included studies, which varied in patient populations, graft types, surgical techniques, and outcome reporting. All outcomes were reported using forest plots with 95% confidence intervals (CIs). An odds ratio analysis was used for dichotomous data assessment, and the mean difference metric was used for continuous variables.

Results

Literature Search Results

**Figure 1:**
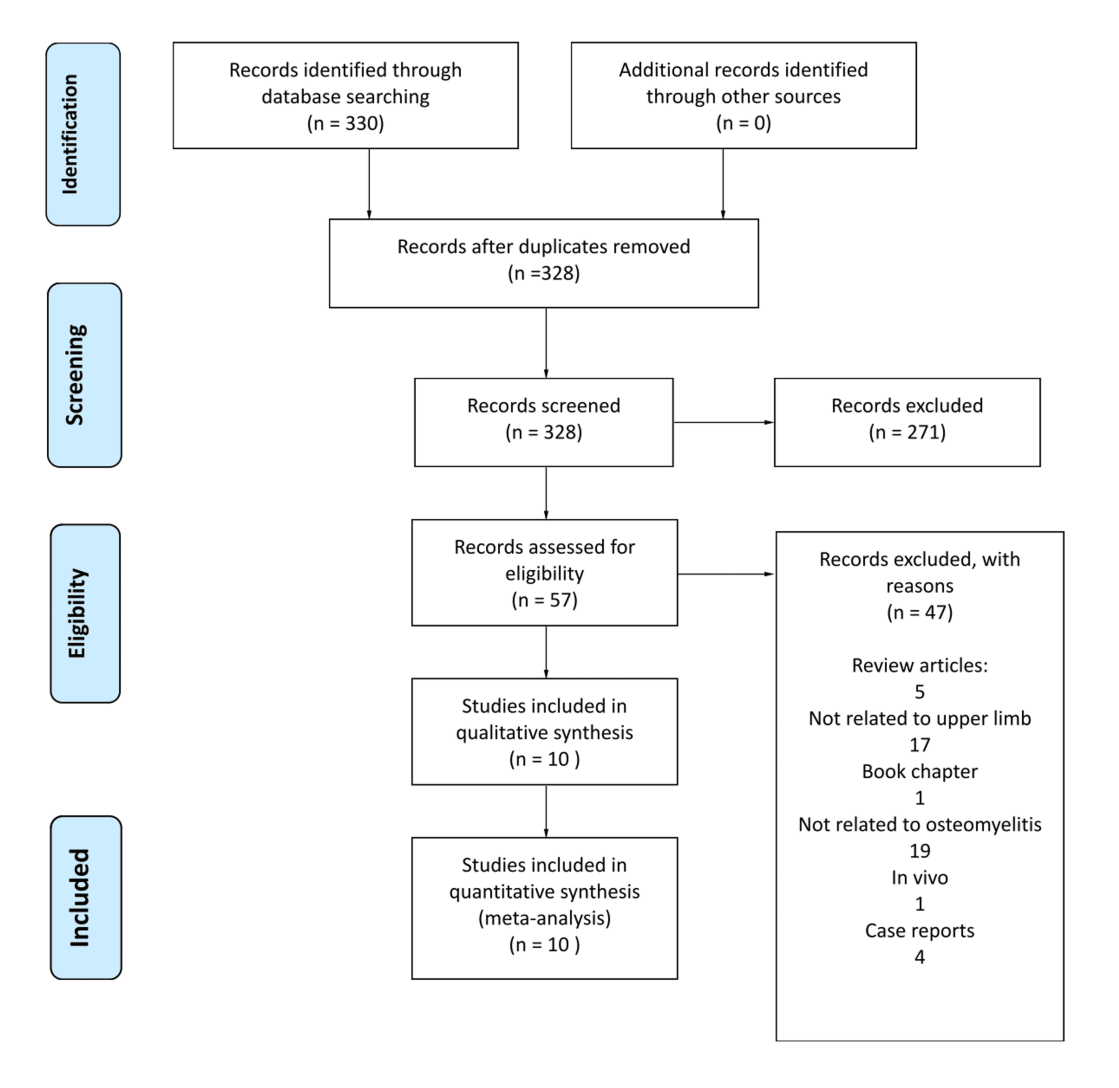
PRISMA chart for the literature search PRISMA: Preferred Reporting Items for Systematic Reviews and Meta-Analyses

As shown in Figure 1, the literature search initially identified 330 studies. After the removal of two duplicates, 328 articles were screened by the authors (ALS and MSB). A total of 271 articles were excluded, with 10 studies meeting the criteria for inclusion in assessing VBGs for upper limb OM.

Study Characteristics

All 10 suitable studies were retrospective case series. The number of patient cases, site of and size of defect, graft site, antibiotic therapy, osteosynthesis method, time to grafting, follow-up period, and reported complications varied across the studies. These are summarised in Table 1.

**Table 1:** Amalgamation of study design, anatomical location of preoperative nonunion, graft site, average defect size (cm), type of transfer, adjuvant treatments (e.g., antibiotics), osteosynthesis methods, average time to grafting from initial injury (months), follow-up period (months), and reported complications FVF: free vascularised fibula; IV: intravenous. ± NA: standard deviations not available or calculable from literature data. Note that studies varied in reporting methods for defect size. Ranges have been provided where individual sizes were not listed, and where mean and standard deviation were either not provided or not calculable from literature data

First author, year	Design	Number of patients	Site of nonunion (preoperative)	Site of graft	Defect size (cm)	Type of transfer	Adjuvant treatment (antibiotics)	Osteosynthesis method	Time from injury to grafting (mean in months)	Follow-up period (mean in months)	Complications
Dell, 1984 [23]	Retrospective case series	4	Forearm	Fibula	5.00-15.00 ± NA	FVF	IV then oral	External fixator, plates, step-cut osteotomies	Not recorded	Not recorded	Not recorded
Mattar, 1994 [25]	Retrospective case series	14	Radius, humerus, ulna	Fibula	6.00-12.00 ± NA	FVF	Not recorded	Wires, plates	Not recorded	39.00 ± NA	Synostosis, vascular thrombosis, nonunion, loss of graft
Yajima, 2004 [18]	Retrospective case series	2	Radius, ulna	Fibula	6.50 ± 2.50	FVF	Local antibiotics	Not recorded	8.50 ± 2.50	Not recorded	None
Kamrani, 2016 [21]	Retrospective case series	7	Radius, ulna	Distal ulna	6.07 ± 2.57	Vascularised pedicle bone graft from the distal ulna	Intravenous antibiotics for 1-3 days followed by oral antibiotics for up to 3 weeks	External fixator, plates, screws	20.71 ± 27.59	25.70 ± NA	None
Mattos, 2017 [24]	Retrospective case series	4	Radius, hand	Femoral condyle	4.00 ± NA	Vascularised medial femoral condyle	6 weeks culture-directed antibiotics	Plates, external fixator	6.40 ± NA	69.50 ± 37.74	Donor site dehiscence
Antonini, 2019 [19]	Retrospective case series	4	Radius, ulna	Fibula	6.75 ± 0.90	FVF	Antibiotic-loaded spacer	Plates	Not recorded	62.00 ± 4.74	None
Ciclamini, 2019 [17]	Retrospective case series	6	Humerus, forearms	Fibula, femoral condyle	10.70 ± 1.60	FVF, pedicled	Nil	Plates, external fixator	Not recorded	48.17 ± 36.65	Nonunion, infection/fistula
Noaman, 2020 [16]	Retrospective case series	15	Radius	Fibula	10.06 ± 1.52	FVF	6.33 months of preoperative treatment, followed by culture-specific antibiotics	Dynamic compression plates	11.06 ± 3.85	33.60 ± 11.55	Stress fracture, fibular head resorption, transient foot drop
Guidi, 2023 [20]	Retrospective case series	4	Radius	Fibula	12.75 ± 6.42	FVF	Not recorded	Plates	Not recorded	Not recorded	Superficial peroneal nerve injury, plate loosening, neuropathic pain
Lefebvre, 2023 [22]	Retrospective case series	10	Humerus	Fibula	14.79 ± 3.18	FVF	6 weeks of IV culture-directed antibiotics	Plates, external fixator, screws	Not recorded	32.30 ± 29.10	Nonunion, elbow stiffness

Studies were included in each meta-analysis only if they reported the relevant outcomes in adequate detail; those with incomplete data or without the necessary individual patient-level information were excluded.

Mean Time to Union

**Figure 2:**
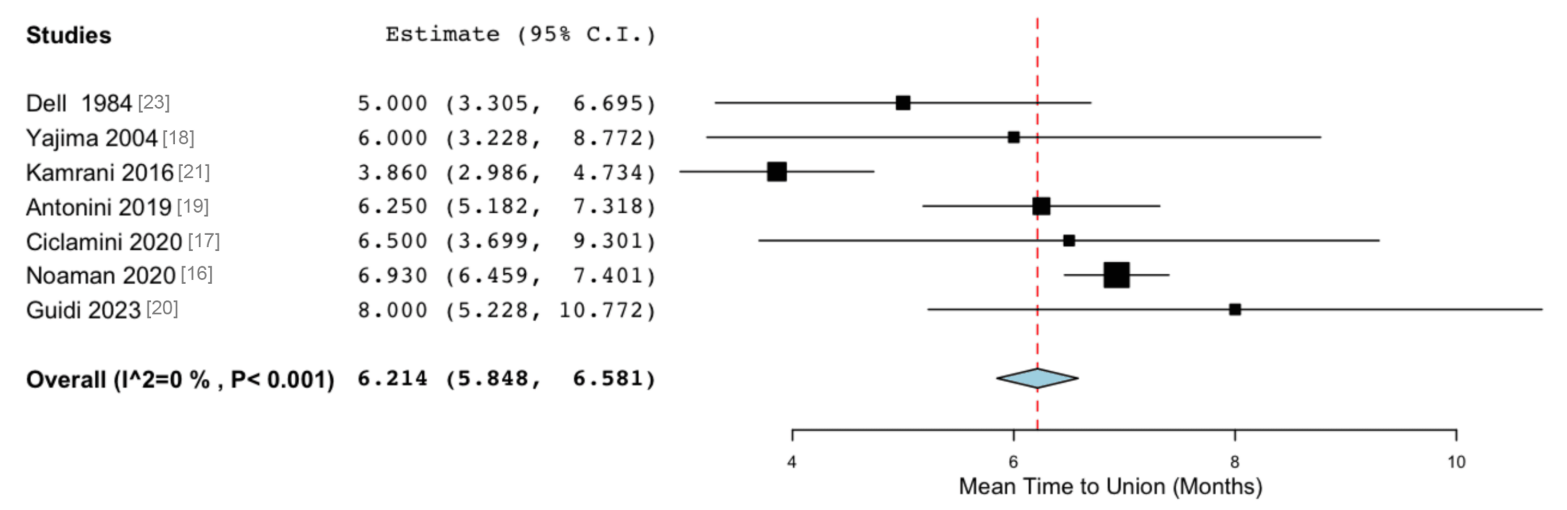
Forest plot showing the time to bone union (months) after the bone grafting procedure. Mean difference analysis: 6.214 months (95% CI 5.848-6.581), standard error 0.187 (p < 0.001). Heterogeneity: I2 = 0%, p < 0.001 CI: confidence interval

Seven studies reported the average time to radiographic bone union. The mean time to union was 6.214 months post-VBG (5.848, 6.581, Figure 2). There was no significant heterogeneity amongst the included studies (I² = 0%, p = 0.997), indicating a high level of consistency in the results.

Stress Fractures

**Figure 3:**
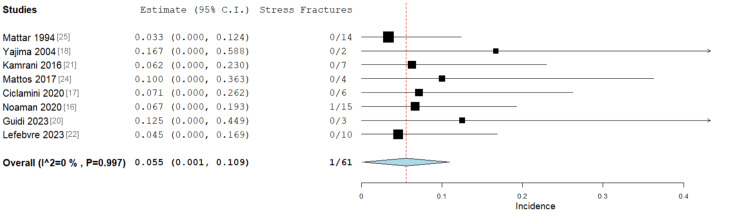
Forest plot showing the rate of patients developing stress fractures after VBG, with an overall incidence of 5.5%. Overall: 0.055 (95% CI 0.001-0.109), standard error 0.028 (p = 0.044). Heterogeneity: I2 = 0%, p = 0.997 VBG: vascularised bone graft; CI: confidence interval

Eight studies examined the incidence of stress fractures in patients who underwent VBG. Of the 61 patients included, only one (1.6%) was noted to have a stress fracture on follow-up [16]. This fracture occurred through the grafted fibula segment after transplantation into a distal radius defect [16]. No study has reported a stress fracture related to the harvest site. The overall incidence of stress fractures was 5.5% (Figure 3). There was no significant heterogeneity amongst the studies (I² = 0%).

Incidence of Union

**Figure 4:**
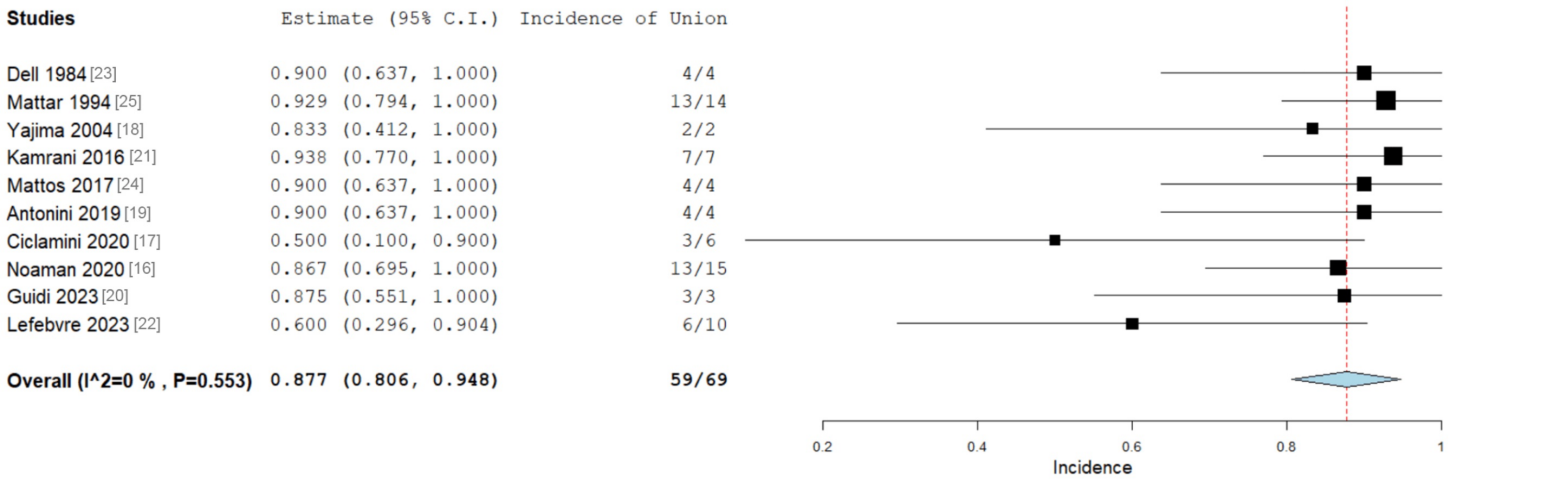
Forest plot showing the incidence of bone union after VBG, with 85.5% (n = 59) of the patients achieving bony union. Overall: 0.877 (95% CI 0.808-0.948), standard error 0.036 (p < 0.001). Heterogeneity: I2 = 0%, p = 0.553 VBG: vascularised bone graft; CI: confidence interval

All 10 studies reported the number of patients with successful bone union after upper limb VBG. One patient was lost to follow-up and was not included in the analysis [20]. This patient showed early signs of consolidation on a three-month postoperative X-ray. Of the 69 patients, 59 (85.5%) achieved bone union after VBG, with an overall incidence of 87.7% (Figure 4). Heterogeneity was low (I² = 0%).

Infection Recurrence

**Figure 5:**
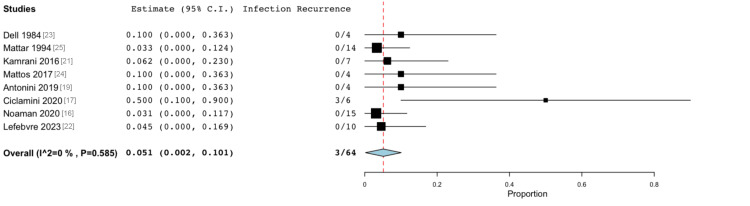
Forest plot showing infection recurrence in 4.69% of patients who developed recurrent infections after bone grafting. Overall: 0.051 (95% CI 0.002, 0.101), standard error 0.025 (p = 0.042). Heterogeneity: I2 = 0%, p = 0.585 CI: confidence interval

In the eight studies that met the inclusion criteria for this outcome, infection recurrence was observed in three (4.69%) of 64 patients after VBG. Following the analysis, the overall incidence of infection recurrence was calculated to be 5.1% (Figure 5), with low heterogeneity (I² = 0%).

Additional Surgery Post Bone Graft

**Figure 6:**
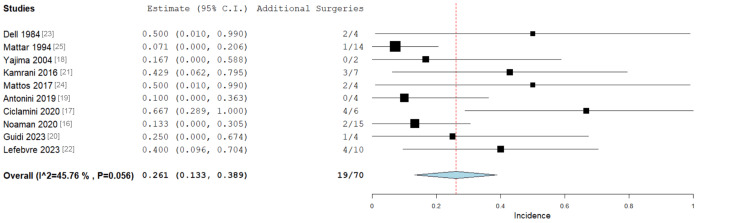
Forest plot showing the number of patients requiring additional surgery post bone graft, with 27.5% requiring additional surgery after the post-bone graft procedure. Overall: 0.261 (0.113-0.389, p < 0.001), standard error 0.065. Heterogeneity: I2 = 45.76%, p = 0.056 CI: confidence interval

All 10 studies reported whether patients required additional surgery after VBG, and 19 (27.5%) patients required these procedures. These included repeat grafting attempts (x2), removal of metalwork, implant revisions (x4), implant removal (x2), resection/shortening of the ulna (x3), closure of a dehisced wound, and necrosectomy. Overall, the incidence of additional surgery after VBG was 26.1% (Figure 6). Moderate heterogeneity was observed across studies (I² = 45.8%), likely reflecting differences in case characteristics such as operative site, defect size, and surgical technique.

Microbial Profile

**Figure 7:**
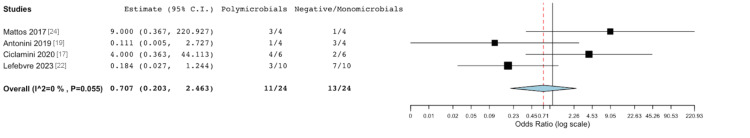
Forest plot depicting a comparison of patients who had polymicrobial infections (>2 pathogens) against those with negative or monomicrobial cultures prior to VBG. Odds ratio: 0.707 (0.203-2.463, p = 0.586), standard error 0.637. Heterogeneity: I2 = 0%, p = 0.055 CI: confidence interval; VBG: vascularised bone graft

Across the four studies that detailed the implicated pathogens, 11 of 24 (45.8%) patients presented with polymicrobial OM prior to VBG. The remaining 13 (54.2%) patients had negative cultures or monomicrobial infections. The odds ratio of polymicrobial versus monomicrobial infection was 0.707 (Figure 7); however, no significant difference was observed between the two groups (p = 0.586). Heterogeneity was low (I² = 0%).

Risk of Bias

All 10 studies scored 3/4 and 3/3 in selection and outcome, respectively, owing to clear case definitions, well-defined treatment outcomes, and adequate follow-up periods (Table 2). However, the lack of stated control for patient demographics and the absence of alternative treatment/control groups reduced the comparability score to 0/3 globally.

**Table 2:** Newcastle-Ottawa scale assessing the risk of bias in the 10 studies [28] Star-rated domains include selection, compatibility, and outcomes, with a greater number of stars representing better methodological quality

First author, year	Selection	Comparability	Outcome
Dell, 1984 [23]	***		***
Mattar, 1994 [25]	***		***
Yajima, 2004 [18]	***		***
Kamrani, 2016 [21]	***		***
Mattos, 2017 [24]	***		***
Antonini, 2019 [19]	***		***
Ciclamini, 2019 [17]	***		***
Noaman, 2020 [16]	***		***
Guidi, 2023 [20]	***		***
Lefebvre, 2023 [22]	***		***

Discussion

OM causes high morbidity and places considerable demand on healthcare resources, indicating that there is significant merit in identifying the most efficacious treatment modalities. Whilst there are several individual cohort studies, a pooled analysis has not yet been conducted. The findings of this study show that VBGs offer significant promise in treating OM of the upper limb, particularly where other treatments have failed.

Bone grafts are classified based on their properties, namely, osteoconductive (wherein they provide scaffolding/structural support for the recipient native bone to grow across), osteoinductive (where they induce native bone cells to proliferate via cellular stimulation), and osteogenic (where they provide both scaffolding and promote native cells to grow across the graft). VBGs, which have a rich circulatory supply, have a greater propensity to be integrated into the host tissue and have more access to systemic antibiotic therapy. In our study, we examined the application of VBGs in the treatment protocol, as it is the standard of care for such surgical interventions. The vascularity of these bone grafts is key to the survival of bone in chronically scarred and infected tissue beds, providing nutrition and bone growth, maintaining an immune response, and making percolation of antibiotics to the tissue bed at the microcellular level possible.

All studies were consistent in reporting the time to union in months. The average time to union following VBG for upper limb OM was 6.214 months (p < 0.001). This is shorter than the mean time to union reported for the same procedure in treating lower limb defects but slightly longer than that reported by Han et al. following humeral defect reconstruction with VBG [29,30]. Other methods of treating infected fracture nonunion have yielded similar results. Patients treated with the implantation of antibiotic calcium sulphate pellets had an average time to union of 5.5 months [31]. Specifically in cases of infected forearm nonunion, the Masquelet method - a two-stage reconstructive procedure in which a temporary cement spacer induces an organic membrane to support a subsequent non-VBG - yields average union times of 7.8 months [32].

The overall incidence of bone union after VBG was 87.7% (p < 0.001), which was higher than the reported rates of union for implantation of antibiotic beads, but lower than that of the Masquelet method [32,33]. It was also higher than the 77% union rate reported by Han et al. for patients undergoing VBG at a previously infected site [29]. High union rates despite recent infection may reflect the benefits of implanting conduit materials with a pre-established blood supply and therefore a richer environment for osteoblast proliferation.

The risk of infection recurrence was 5.1% (p < 0.042). This is lower than the recurrence rates reported for the treatment of bone and joint OM, which stand at approximately 13%-14% according to the OVIVA trial [2]. It is also much lower than the 18.3% reported by Han et al. following VBG for OM (of both upper and lower limbs) [29]. Of note, all three infection recurrences amongst our dataset occurred within the cohort of a single constituent paper [17]. Unlike more common outcomes such as bone healing, the relative rarity of infection recurrence makes its incidence harder to predict reliably. It is difficult to determine whether these three infection recurrences reflect outcomes for a specific centre or for the VBG procedure as a whole.

Stress fractures are also a recognised complication of VBG, occurring both at the graft itself and in bones related to the harvest site [29,34]. In this study, a stress fracture occurred in only one out of 61 included patients, with an overall incidence of 5.5% (p = 0.044). This is considerably lower than incidences reported in other literature, which range from 11.5% to 13.1% [35]. The stress fracture in this analysis occurred at the VBG itself, and there were no reported cases of stress fractures related to the graft donor site [16].

Multiple procedures are often required to gain adequate control of established bone infections, as was the case for 19 (26.1%) patients in this analysis (p < 0.001). Subsequent procedures included repeat grafting attempts, metalwork removal, implant revisions, dehisced wound closure, and necrosectomy. Notably, repeat operations were required for both patients who underwent single-stage VBGs and those who underwent two-stage initial VBG procedures. This is in keeping with existing literature, which suggests that there is no significant difference in long bone OM cure rates when comparing single- and two-stage management [36].

Fewer than half of the included studies reported the specific infectious aetiology of individual cases, with only four of 10 suitable for meta-analysis of this secondary outcome. Across these four studies, the incidence of polymicrobial (versus monomicrobial) infection was 70.7%, but failed to reach statistical significance (p = 0.586). This could reflect differences in sampling techniques amongst the respective centres, the high sensitivity of microbial culturing, or skin commensal contamination. The studies did not report specific sampling or culture methods.

Limitations

Our findings demonstrate the efficacy of VBGs in managing upper limb OM; however, to reliably contrast and compare with the outcome profile for other surgical modalities, an analysis of randomised controlled trials (RCTs) is required. Currently, this form of evidence is lacking in the literature. In the interim, further studies should perform a multiple-arm analysis of the more ubiquitous retrospective case series for each modality.

Most of the included studies also featured mixed patient profiles, requiring segregation of the requisite cases for an analysis focused solely on upper limb OM. Whilst the study ultimately achieved a well-sized cumulative cohort of patients, this did yield some heterogeneity between studies. Future analyses would benefit from the use of larger published case series or RCTs, and a greater number of studies should focus solely on VBGs in the context of upper limb OM.

## Conclusions

This meta-analysis demonstrates that VBGs constitute an effective treatment option for upper limb OM, yielding outcomes that are comparable to and, in some outcomes, more favourable than those associated with other treatment modalities. The majority (n = 59, 87.7%) of patients achieved post-procedural bone union at an average of 6.2 months post-op. The incidence of postoperative stress fractures (5.5%) and infection recurrence (5.1%) was significantly lower than that reported in other studies. Although this analysis utilised only a modest number of individual patient cases, all but one measured outcome reached statistical significance, providing strong evidence for VBG efficacy.

The authors advocate further studies to corroborate these positive findings on a larger scale. Prospective RCTs comparing VBGs to other recognised treatment modalities would offer robust evidence to further advise best clinical practice.
